# COPD in Nonsmokers: Reports from the Tunisian Population-Based Burden of Obstructive Lung Disease Study

**DOI:** 10.1371/journal.pone.0151981

**Published:** 2016-03-24

**Authors:** Meriam Denguezli, Hager Daldoul, Imed Harrabi, Louisa Gnatiuc, Sonia Coton, Peter Burney, Zouhair Tabka

**Affiliations:** 1 Laboratory of Physiology, Faculty of Medicine Ibn El Jazzar, Sousse, Tunisia; 2 Department of Epidemiology, University Hospital Farhat Hached, Sousse, Tunisia; 3 National Heart and Lung Institute, Imperial College London, Royal Brompton Campus, London, United Kingdom; Universität Bochum, GERMANY

## Abstract

**Background:**

It’s currently well known that smoking and increasing age constitute the most important risk factors for chronic obstructive pulmonary disease (COPD). However, little is known about COPD among nonsmokers. The present study aimed to investigate prevalence, risk factors and the profiles of COPD among nonsmokers based on the Tunisian Burden of Obstructive Lung Disease (BOLD) study.

**Methods:**

807 adults aged 40 years+ were randomly selected from the general population. We collected information about history of respiratory disease, risk factors for COPD and quality of life. Post-bronchodilator spirometry was performed for assessment of COPD. COPD diagnostic was based on the post-bronchodilator FEV_1_/FVC ratio, according to the Global Initiative for Obstructive Lung Disease (GOLD) guidelines. The lower limit of normal (LLN) was determined as an alternative threshold for the FEV_1_/FVC ratio.

**Results and Conclusions:**

Among 485 nonsmokers, 4.7% met the criteria for GOLD grade I and higher COPD. These proportions were similar even when the LLN was used as a threshold. None of the nonsmokers with COPD reported a previous doctor diagnosis of COPD compared to 7.1% of smokers. Nonsmokers accounted for 45.1% of the subjects fulfilling the GOLD spirometric criteria of COPD. Nonsmokers were predominately men and reported more asthma problems than obstructed smokers. Among nonsmokers significantly more symptoms and higher co-morbidity were found among those with COPD. Increasing age, male gender, occupational exposure, lower body mass index and a previous diagnosis of asthma are associated with increased risk for COPD in nonsmokers. This study confirms previous evidence that nonsmokers comprise a substantial proportion of individuals with COPD. Nonsmokers with COPD have a specific profile and should, thus, receive far greater attention to prevent and treat chronic airway obstruction.

## Introduction

Chronic obstructive pulmonary disease (COPD) represents a major public health problem and one of the leading causes of morbidity and mortality worldwide. The population prevalence of COPD is growing in developing countries and especially in North Africa [1–2]. Among the major causes of COPD, the role of tobacco smoking is well recognized and accordingly, recent studies of COPD have been largely focused on the smoking rather than the non-smoking population [3]. However, there is current evidence that never smokers can also develop chronic airflow obstruction and might thus comprise a substantial proportion of this disorder [4]. According to a recent review of the American Thoracic Society (ATS), a substantial proportion of COPD cases are not associated with smoking, especially among women and residents of developing countries [5]. Several factors have been implicated such as occupational exposure and respiratory problems in childhood as causes of airways obstruction. Outdoor air pollution, second hand smoke, biomass smoke and chronic asthma are leading causes of lung function decrement and irreversible airway obstruction among nonsmokers. This problem is more prevalent in developing countries compared with the developed ones [5,6,7]. However, our understanding of these risk factors for the development of chronic non-reversible airway obstruction in nonsmokers is still incomplete and only a few studies have described this population in greater details [8–9]. Therefore, insights into the epidemiology of COPD in nonsmokers may be of interest.

To investigate the prevalence and risk factors of COPD among nonsmokers, we used the data of the population-based Tunisian Burden of Obstructive Lung Disease (BOLD) study. We reported the prevalence of COPD in nonsmokers and described the clinical presentation and the severity of the disease and particularly associated risk factors.

## Materials and Methods

This study was approved by the ethics committee of the Medical School of Sousse and written informed consent was obtained from all the participants.

### Study population

We followed the BOLD protocol as it has been described elsewhere [10,11,12]. The survey was conducted on a gender-stratified representative random sample of civilian, non-institutionalized residents selected from the general population living in the urban area of Sousse (Tunisia).

Two quartiers with clear administrative boundaries were selected for convenience (Erriadh and Jawhara) and districts were sampled at random from each of the two selected quartiers (9 districts from Erriadh and 8 districts from Jawhara). Households were then sampled from each selected district and all the individuals aged 40 years or over living in the selected households were invited to participate in the study.

### Outcome measurements

Spirometry was performed according to the American Thoracic Society (ATS) criteria by trained and BOLD-certified technicians who performed spirometry on participants before and 15 minutes after administering 200 μg of salbutamol (Ventolin; GlaxoSmithKline,Middlesex, UK) via metered-dose inhaler with a one-way valve volumatic spacer (Volumatic; GlaxoSmith-Kline; Research Triangle Park, NC) [13]. Portable spirometers (EasyOnendd.Medizintechnik; Zurich, Switzerland) were used in this study and were checked for calibration daily, using a 3.00-liter syringe. All spirometry data were reviewed and graded for quality by the BOLD Pulmonary Function Quality Control Center. The quality of spirometry maneuvers was monitored regularly, and technicians whose quality scores dropped below pre-specified levels were retrained. Acceptability and reproducibility were assessed according to the ATS/ERS 2005 criteria as described by Enright and colleagues [14]. Only tests that met high quality scores were used for the final analysis.

### Questionnaires data

The questionnaires data were obtained by trained and certified interviewers who conducted face-to-face interviews in the participant’s native language. The questionnaires used in this study assessed data on the medical history and disease symptoms, risk behaviors for COPD, co-morbidities, respiratory diagnoses and activity limitation [12].

### Definitions

According to the Global Initiative of Lung Disease (GOLD) guidelines, COPD was defined as a post-BD FEV_1_/FVC < 0.7 [15]. COPD stages were categorized as: Stage I: if FEV_1_ ≥ 80% predicted; Stage II: if FEV_1_ ≥ 50 and < 80% predicted; Stage III: if FEV_1_ ≥ 30 and < 50% predicted; and Stage IV: if FEV_1_ < 30% predicted [15–16]. The third US National Health and Nutrition Examination Survey (NHANES III) was used for computing predicted values and lower limits of normal (LLNs) [17].

Nonsmokers referred to subjects who had smoked on average <1 cigarette/day for <1 year or had never smoked, ex-smokers were subjects who had ceased smoking at least 12 months prior to the interview and ever smokers (current or former smokers) were defined as persons who had smoked > 20 packs of cigarettes in a lifetime or > 1 cigarette/day for a year. Nonsmokers exposed to COPD risk factors were grouped and their data analyzed to determine the specific characteristics of COPD in these subjects in comparison to smokers with COPD.

The following information were also collected: Body mass index (BMI) value (Kg/m^2^), number of years of education, hospitalization for breathing problems prior to the age of 10 years, respiratory symptoms (cough, phlegm, wheezing, and dyspnoea), physician diagnosis of asthma, emphysema, COPD or tuberculosis and comorbidity (heart disease, hypertension, diabetes).

Family history of obstructive airway disease was defined as an affirmative answer to whether the participant reported a first-degree relative with asthma, chronic bronchitis, emphysema or COPD.

The use of bronchodilating treatment was considered positive if the subject affirmed past or current use of any medication for the treatment of bronchoconstriction.

Exposure to environmental tobacco smoke during childhood was considered positive if a household member smoked cigarettes in the home during the subject’s childhood.

Current exposure to cigarette smoke was reported separately for the home and the work place. Exposure at home was present if a person in the household had smoked cigarette, pipe, or cigar during the past two weeks. Exposure at work was present if the participant smelled tobacco smoke at the workplace for more than 4h/day.

To assess long-term occupational exposure to airway irritants, participants were asked whether they had worked ≥3 months in one of the following three categories of occupations: (1) organic dust (farming; flour-, feed-, or grain-milling; cotton- or jute-processing; forestry- or wood-milling; and fish-processing); (2) inorganic dust (asbestos, aluminum, coal, or hard-rock mining; tunneling, foundry, or steel-milling; and sandblasting); (3) irritant gazes, fumes or vapors (through welding, fire fighting, chemical or plastic manufacturing, public transportation, and dry-cleaning chemicals).

Measures of biomass exposure were based on self-reported responses indicating if participants had experienced at least 6 months’ exposure to indoor open fire using coal, coke, wood, crop residue or dung.

### Statistical analysis

BOLD participants who completed the study questionnaires and had acceptable postbronchodilator spirometry measures were included in the present analysis. Bivariate comparisons were performed using Student T test to compare continuous measures across groups and χ^2^ tests to compare categorical measures.

Calculations of odd ratios (ORs) and 95% CI values for COPD in relation to potential risk factors were performed with multivariate logistic regression models. Models included gender; age category (40–49, 50–59, 60–69 and 70+ years); years of school; occupational exposure to gases, vapors or fumes; and indicators for ≥10years of exposure to cooking biomass and heating biomass; passive-smoking exposure; hospitalization for breathing problems during childhood; self-reported physician-diagnosed conditions including asthma, cardiovascular disease, hypertension or diabetes and BMI category (<20, 20–25, 25–30, 30–35 and ≥35). All statistical tests were performed with IBM SPSS statistics software (version 20), and a P value of <0.05 was considered statistically significant.

## Results

### Sample description

Of the 807 subjects sampled from Sousse region in Tunisia, 717 responders were included in the final analysis. Responders were randomly included to the study and the response rate was 90%. 77 non-responders and 13 ineligible participants were excluded from the study for the following reasons: refusals, contact failures, spirometry ineligibility, and failed attempts. Among the 717 interviewees, 56 failed to complete the spirometry testing and 661 completed acceptable and reproducible post-BD spirometry and questionnaires. There were no significant differences in age, sex and smoking status between responders and non-responders, suggesting that the study participants are highly representative of the general population “Table 1”.

**Table 1:** Comparison of responders and non-responders.

	Responders N (%)	Non responders N (%)	P-value*	X2-value
**Gender**			0.752	15.47
Male	331 (46.16)	37 (48.05)		
Female	386 (53.83)	40 (51.94)		
**Age group, yr**			0.805	7.23
40–49	263 (36.68)	25 (32.47)		
50–59	290 (40.44)	33 (42.86)		
60–69	118 (16.45)	15 (19.48)		
70+	46 (6.41)	4(5.19)		
**Smoking status**			0.597	25.86
Current smoker	196 (27.33)	20 (25.97)		
Nonsmoker	521 (72.66)	57 (74.02)		
**Total**	717 (90.99)	77 (9.01)		

* Two-sided p-value based on Pearson chi-square test.

Responders are subjects that answered the core questionnaire and performed post BD spirometry regardless of quality.

Among all participating subjects, nonsmokers accounted for 73.4% (n = 485), of whom 32.8% were males and 67.2% were females. Nonsmokers were more likely to be women in all age groups while current smokers were predominately males. Study subjects with more than 20 pack-years of smoking history added up to 24.4%. Characteristics of the study population are summarized in Table 2.

**Table 2:** Population characteristics for smokers and nonsmokers.

	Men (n = 309)		Women (n = 352)	
Characteristics	Nonsmokers	Smokers	Nonsmokers	Smokers
Subjects, No. (%)	159 (24)	150 (22.7)	326 (49.3)	26 (4)
Age, mean (SD), y	55 (9)	51 (9)	52 (8)	50 (8)
Age category, No. (%)				
40–49	28 (9.1)	88 (28.5)	117 (33.2)	16(4.5)
50–59	28 (9.1)	90 (29.1)	140 (39.8)	13 (3.7)
60–69	18(5.8)	39(12.6)	46 (13.1)	2 (0.6)
≥70	4 (1.3)	14(4.5)	17 (4.8)	1 (0.3)
BMI, mean (SD) kg/m^2^	29.14 (12.06)	25.54 (4.61)	31.45 (5.64)	28.89 (5.42)
Education, mean (SD), y	3.43 (1.46)	3.40 (1.43)	2.45 (1.31)	2.92 (1.09)
Smoking,>20 pack-years, No. (%)	-	36 (11.7)	-	7 (2)

Data are presented as % unless otherwise indicated. Abbreviations: BMI, Body mass index

### Respiratory symptoms in nonsmokers

#### Prevalence of non-reversible airway obstruction in nonsmokers

**Fig 1:**
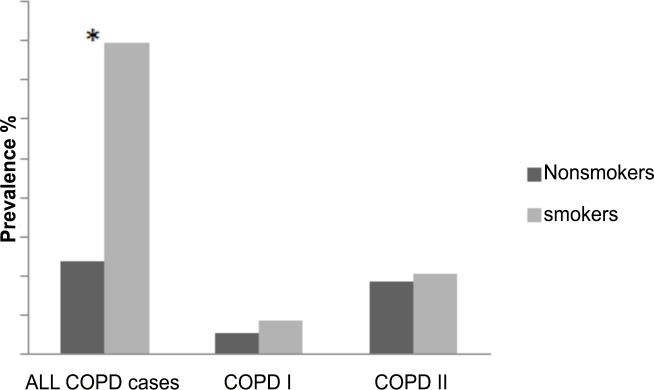
Prevalence (%) of COPD in smokers and nonsmokers respectively by disease severity. COPD: Chronic Obstructive Pulmonary Disease; GOLD I: Global Initiative for Chronic Obstructive Lung Disease Stage I COPD; *: p<0.01, Significant difference between smokers and nonsmokers.

In nonsmokers, the prevalence of non-reversible airway obstruction (GOLD grade I or higher COPD) was 4.7% compared to 15.9% among current smokers (X2 = 22.61, p<0.01). 3.7% of nonsmokers and 4.1% of smokers met the criteria for GOLD grade II COPD (Fig 1). Of all the subjects fulfilling the GOLD spirometric criteria of airway obstruction, nonsmokers accounted for 45.1%: 38.4% of all GOLD grade I cases and 47.4% of all GOLD grade II cases. Overall, the prevalence of GOLD grade I+ COPD was 7.8% (1.2) and of GOLD grade II COPD was 5.7% (1.1).

When the LLN was used as a threshold for the FEV_1_/FVC ratio instead of the fixed ratio of 0.7, prevalence of airway obstruction among nonsmokers (FEV1/FVC < LLN and FEV1< 80% predicted) was similar to that found using the GOLD fixed ratio (3.1% (1.2) vs 3.5% (1.5) respectively).

**Fig 2:**
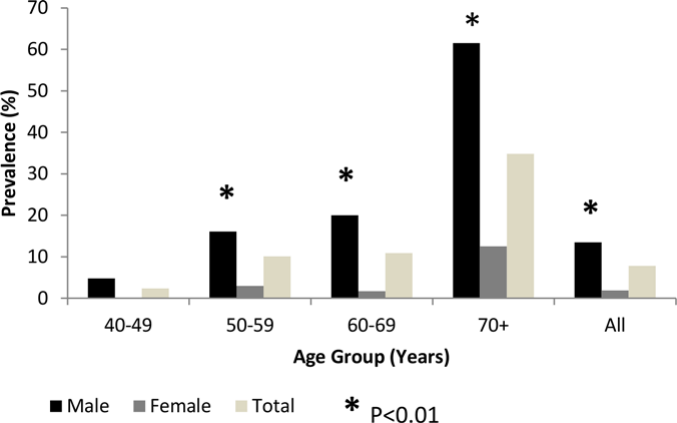
Prevalence of COPD (GOLD I and higher COPD) by gender and age groups. Chronic Obstructive Pulmonary Disease; GOLD I: Global Initiative for Chronic Obstructive Lung Disease Stage I COPD; *: p<0.01, Significant difference between male and female according to age.

The prevalence of GOLD grade I+ COPD increased with age in both sexes but was statistically significant only in the male group (p<0.01). For each age group, the prevalence of GOLD grade I+ COPD was greater in men than in women (X2 = 57.85, p<0.01) (Fig 2).

**Fig 3:**
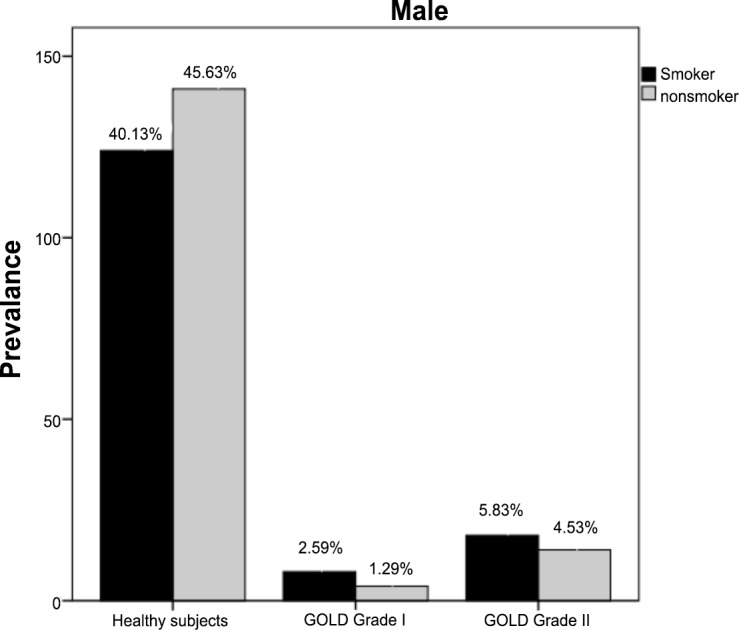
Population characteristics of male smokers and nonsmokers. COPD: Chronic Obstructive Pulmonary Disease; GOLD I: Global Initiative for Chronic Obstructive Lung Disease Grade I COPD; GOLD II: Global Initiative for Chronic Obstructive Lung Disease Grade II COPD.

**Fig 4:**
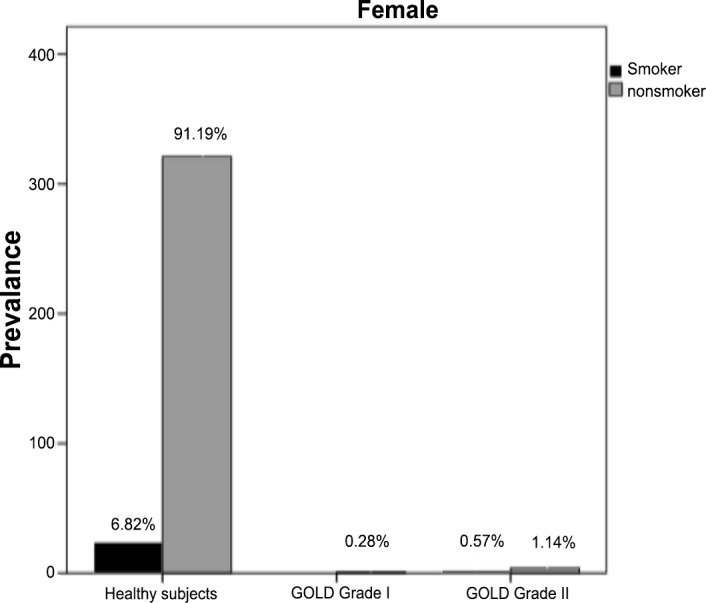
Population characteristics of female smokers and nonsmokers. COPD: Chronic Obstructive Pulmonary Disease; GOLD I: Global Initiative for Chronic Obstructive Lung Disease Grade I COPD; GOLD II: Global Initiative for Chronic Obstructive Lung Disease Grade II COPD.

Among smokers, COPD prevalence was generally higher in men than in women (8.41% versus 0.5%, X2 = 35.76, p<0.01). The same trend was noticed for nonsmokers and the prevalence of both GOLD grade I and grade II COPD was significantly higher in males than in females (1.3% versus 0.3% and 4.5% versus 1.1% respectively; X2 = 22.73, p<0.05) (Figs 3 and 4). Nonsmokers with GOLD grade II COPD had approximately the same age than smokers.

In smokers, as expected, prevalence of COPD (GOLD grade I and II) increased with increasing pack-years of cigarette smoking in both men and women. The prevalence of airflow obstruction in subjects with GOLD grade I COPD increased from 3.5% in non smoking subjects to 16.1% in those with a smoking history ≥ 20 pack-years. Similarly, the prevalence of GOLD grade II COPD increased from 2.7% in non-smoking subjects to 8.2% in those with the most pack-years of smoking.

#### Clinical profile of COPD in nonsmokers

Males accounted for the majority of non-smoking COPD subjects. Among subjects with GOLD grade I+ COPD, there was no significant difference between smokers and nonsmokers in age, BMI, education, FEV1/FVC ratio, FEV1% predicted values, family history of obstructive airway diseases, physician diagnosis of emphysema, history of exposition to passive smoking during childhood, exposition to biomass smoke for heating and percentage and duration of occupational exposure to dusts/gases/fumes “Table 3”.

**Table 3:** Characteristics of chronic obstructive pulmonary disease (COPD) among nonsmokers compared with those in smokers.

	Smoking COPD	Non-smoking COPD	*P*-value
**Characteristics**			
Subjects, No. (%)	28 (55)	23 (45)	
Age, mean (SD), y	61.39 (9.04)	62.74 (11.23)	0.252
Sex, female	7.1%	21.7%	<0.01
BMI, mean (SD), Kg/m^2^	24.16 (5.01)	29.27 (4.98)	0.801
Education, mean (SD), y	8.43 (4.88)	8.61 (4.78)	0.892
FEV1% pred, mean (SD)	50.10 (15.65)	46.46 (13.89)	0.295
FEV_1_/FVC %, mean (SD)	61.31 (9.37)	61.14 (9.07)	0.981
Hospitalized for breathing problems<age of 10	0%	4.34%	0.02
Family history of obstructive airway diseases	3.57%	4.34%	0.755
Doctor-Diagnosed COPD	7.1%	0%	<0.01
Use of medication for respiratory disease	21.4%	17.4%	<0.01
Previous contact with health care due to respiratory symptoms	32.1%	39.1%	<0.01
**Respiratory symptoms**			
Chronic sputum production	57.1%	39.1%	0.01
Chronic cough	50%	21.7%	0.03
Wheezing	50%	47.8%	<0.01
Dyspnoea	53.2%	50%	0.01
**Reported co-morbidities**			
Asthma	14.3%	30.4%	<0.01
Heart disease	0%	17.4%	0.021
Hypertension	21.4%	43.5%	0.025
Diabetes	7.1%	17.4%	0.494
Chronic bronchitis	10.7%	13%	<0.01
Emphysema	3.6%	4.3%	0.332
TB	0%	0%	-
**Reported exposures**			
Passive smoking at home	14.3%	21.7%	0.011
Exposition to passive smoking during childhood	64.3%	52.2%	0.631
Indoor open fire with biomass for cooking	21.4%	39.1%	0.007
Indoor open fire with biomass for heating	53.6%	65.2%	0.283
Gases/fumes/vapors in the workplace	64.3%	65.2%	0.554

Data are presented as % unless otherwise indicated. Abbreviations: BMI, Body mass index; FEV_1_: Forced expiratory volume in one second; FVC: Forced vital capacity, %pred: percentage predicted.

Unlike those with smoking related COPD, nonsmoking COPD patients were less likely to have chronic sputum production, cough, wheezing, dyspnoea and to use medications for respiratory disease (X2 = 17.10, p<0.05) while more likely to have received a physician diagnosis of asthma and to have suffered from respiratory diseases during their childhood (X2 = 82.84 p<0.01). Nonsmoking GOLD grade I+ COPD patients also reported a significantly higher rate of co-morbidities than smokers with COPD and than healthy nonsmokers (X2 = 7.65, p<0.05) “Table 3”.

Surprisingly, nonsmoking women with airway obstruction produced significantly more sputum and suffered more frequently from chronic cough than smoking women (13.9% vs 0% and 12% vs 0%, respectively) (X2 = 7.23, p<0.05). This difference could be explained by the healthy smoker effect since smokers that don’t quit most often are those without symptoms.

When comparing nonsmokers with versus without airflow obstruction significantly more symptoms and higher co-morbidity were found in the first group “Table 4”. Indeed, chronic sputum production, wheezing, dyspnoea, current use of medication for obstructive airway disease and respiratory care utilization tended to be significantly more frequent in nonsmokers with GOLD grade I+ COPD compared to those without. In addition, a prior diagnosis of asthma, heart disease, hypertension and chronic bronchitis but not emphysema, was significantly more common among nonsmokers with GOLD grade I+ COPD compared to nonsmokers with normal lung function “Table 4”. Current or prior use of medication for obstructive airway disease was significantly more common among nonsmokers with COPD compared to those without airflow obstruction but also significantly lower compared to smoking COPD patients. Of nonsmokers with GOLD garde I+ COPD, 30.4% had received a prior diagnosis of asthma and 13% had a prior diagnosis of chronic bronchitis and these results were significantly different from those of the healthy nonsmokers (X2 = 27.04, p<0.01). However, among smokers with the same disease, only 14.3% had received a prior diagnosis of asthma and 10.7% a prior diagnosis of chronic bronchitis “Table 4”. Moreover, nonsmoking patients with a grade II COPD received significantly more physician diagnosis of asthma than those with a grade I COPD (X2 = 12.2, p<0.01).

**Table 4:** Respiratory symptoms, co-morbidity, family history of obstructive airway disease, health care consumption and previous physician diagnosis of airway disease in nonsmokers with and without non-reversible airway obstruction.

	FEV_1_/FVC<0.7	FEV_1_/FVC>0.7	*P*-value
**Characteristics**			
Age, mean (SD)	62.74 (11.23)	53.22 (8.64)	0.075
Sex, female	21.7%	69.5%	<0.01
FEV_1_/FVC (%)	61.14 (9.07)	79.74 (5.17)	<0.01
BMI (Kg/m^2^)	29.27 (4.98)	30.76 (8.5)	0.715
FEV1 (% pred)	46.46 (13.89)	70.30 (12.99)	0.845
Education≥12 years	30.4%	26.6%	0.687
Hospitalized for breathing problems<age of 10	4.3%	2.8%	0.750
Doctor-Diagnosed COPD	0%	0.4%	0.752
Family history of obstructive airway diseases	4.3%	6.7%	0.656
Use of medication for respiratory disease	17.4%	4.8%	<0.01
Previous contact with health care due to respiratory symptoms	39.1%	10%	<0.01
**Respiratory symptoms**			
Chronic sputum production	39.1%	23.4%	0.085
Chronic cough	21.7%	24.9%	0.732
Wheezing	47.8%	20.8	<0.01
Dyspnoea	50%	10.6%	0.019
**Reported co-morbidities**			
Asthma	30.4%	8.9%	<0.01
Heart disease	17.4%	5.6%	0.022
Hypertension	43.5%	23.2%	0.026
Diabetes	17.4%	11.7%	0.411
Chronic bronchitis	13%	2.4%	<0.01
Emphysema	4.3%	1.3%	0.231
TB	0%	0%	-
**Reported exposures**			
Passive smoking	21.7%	40.7%	0.07
Indoor open fire with biomass for cooking	39.1%	17.3%	<0.01
Indoor open fire with biomass for heating	65.2%	50.2%	0.247
Irritant gases/fumes/vapors in the workplace	65.2%	51.3	0.192

Data are presented as % unless otherwise indicated. Abbreviations: BMI, Body mass index; FEV_1_: Forced expiratory volume in one second; FVC: Forced vital capacity, %pred: percentage predicted.

### Reported doctor-diagnosed chronic airway disease

In both smokers and nonsmokers with COPD, the prevalence of reported doctor-diagnosed chronic bronchitis, emphysema or COPD was far lower than that determined by spirometry. Indeed, none of the nonsmokers with COPD had ever received a previous doctor diagnosis of chronic bronchitis, emphysema or COPD compared to 7.1% of smokers (X2 = 20.81, p<0.01). In addition, only 39.1% of the nonsmokers and 32.1% of smokers with airflow obstruction had ever been tested by lung function tests (spirometry) (X2 = 28.52, p<0.01). The self-reported doctor-diagnosed COPD prevalence was higher in the smoking females than males and no clear trend was seen between the prevalence of doctor-diagnosed chronic bronchitis, asthma, emphysema or COPD and the smoking subjects’ age.

### Risk factors for COPD among nonsmokers

We performed univariate and multivariate logistic regression to assess the association of COPD with gender, age, education, exposure to occupational dusts/gases/fumes, to biomass smokes and to passive smoking, childhood history of respiratory disease, comorbidities diagnosis and BMI “Table 5”. After mutual adjustment for all these potential factors in the model, we found that in our study population, the most predominant risk factor for airway obstruction among nonsmokers was increasing age. Indeed, COPD was significantly more common in subjects aged 70+ years (OR = 21.61, p = 0.001) compared to subjects aged 40–49 years. Male gender and occupational exposure to dusts/gases/fumes were also associated with an increased likelihood of developing stage I+ COPD, whereas biomass smokes exposure for cooking and heating and less education were not associated with a risk of developing this disease. Moreover, a previous diagnosis of asthma and a BMI<20kg/m^2^ constitute independent risk factors for stage I+ COPD. However, in our data, education, area of domicile, socio-economic status and family history of obstructive airway disease were not found to constitute significant risk factors for non-reversible airway obstruction.

**Table 5:** Independent predictors of GOLD stage I+ COPD in nonsmokers: Multivariate logistic model.

Variable	OR	95% CI	P Value
**Gender**			
Male	14.837	2.626–83.836	0.002
Female	Reference	-	-
**Age category, y**			
40–49	Reference	-	-
50–59	3.046	0.500–18.549	0.227
60–69	3.612	0.514–25.362	0.197
≥70	57.769	6.317–528.327	<0.01
**Education, y**			
1-y increase	1.996	0.396–10.054	0.402
**High-risk occupation, y**			
≤10-y exposure	0.781	0.116–5.240	0.799
≥10-y exposure	1.87	1.140–12.863	0.015
**Biomass fuel**			
≥ 10-y cooking	1.479	0.373–5.855	0.578
≥ 10-y heating	0.760	0.198–2.921	0.690
**Passive smoking**			
Exposed	0.546	0.193–1.545	0. 254
**Childhood hospitalisation**			
Yes	3.075	0.350–27.017	0.311
**Comorbidities, diagnosis**			
HD/HT/DM	1.758	0.585–5.282	0.315
Asthma	10.621	2.897–38.937	<0.01
**BMI, Kg/m**^**2**^			
BMI<20	4.010	1.31–12.19	0.018
20≤BMI<25	Reference	-	-
25≤BMI<30	0.134	0.009–1.937	0.140
30≤BMI<35	0.165	0.011–2.466	0.192
BMI≥35	0.107	0.006–2.075	0.140

Abbreviations: DM = diabetes mellitus; HD = Heart disease; HT = hypertension; BMI, Body mass index.

## Discussion

The present study is the first to provide population-based estimates of the prevalence of airflow obstruction in nonsmokers in Tunisia as defined by LLN, FEV1/FVC ratio and FEV_1_<0.70 predicted.

There were three main findings in this study: 1) our data confirm previous evidence that a substantial proportion of individuals with COPD are nonsmokers and that they are usually under diagnosed with the disease. 2) More than two-thirds of nonsmokers with mild to moderate airway obstruction are men. 3) The most predominant risk factors for airway obstruction among nonsmokers include advanced age, gender, occupational exposure, lower body mass index and a previous diagnosis of asthma. This analysis of BOLD data in Tunisia also shows that, nonsmokers accounted for almost the half (45%) of COPD cases. This proportion is higher than described in other population based studies from developed countries like UK (22.9%) and Spain (23.4%), but similar to the proportions reported among the COPD cases in the United States (25%) and in China (38.6%) [18–19].

In our study population, 3.5% of the nonsmokers have at least stage I COPD. As with results from other studies which used post-bronchodilation GOLD criteria to define and grade COPD, prevalence of airflow obstruction in Tunisian nonsmokers appears in the lower range both for males and females [20,21,22]. This finding remained valid when the fixed FEV1/FVC ratio was used instead of the LLN. However, in comparison to other studies using the same diagnostic criteria in the same age groups our results were similar to those found in Switzerland (3.4%) [23], in China (4%) [24], in Germany (3.7%), in Canada (3.5%) and in Sweden (3.4%) [22].

COPD does not develop suddenly, but rather exposure to risk factors over a considerable period of time. Increasing age, in our population, is a strong risk factor for having COPD, indeed, the prevalence of COPD increases with age in both smokers and nonsmokers and was significantly higher in people ≥70 years of age. These findings are consistent with the results of other countries using the same BOLD methodology [22–24] and in many other previous epidemiological studies who showed a steep gradient in COPD prevalence with increasing age in both smokers and nonsmokers [10,20,25,26,27].Using a fixed FEV_1_/FVC ratio < 0.70 as a cut point for airflow obstruction constitutes an important limitation and may lead to overestimation of the disease prevalence in elderly nonsmokers since this ratio has a significant age-related regression [28,29,30].

Nonetheless, using the LLN criterion in the evaluation of FEV_1_/FVC could be an alternative to minimize the potential misclassification [31–32]. Indeed, several previous studies showed that the use of the LLN criterion instead of the fixed ratio criterion minimizes known age biases and better reflects clinically significant irreversible AO [28–30]. However, recent study has demonstrated that individuals with FEV_1_/FVC ratio below 0.7 and above 5th percentile have indeed increased risk of COPD-related death or hospitalization [33]. The latter finding in part justifies the using of a fixed FEV_1_/FVC ratio especially in epidemiological studies (such as BOLD study), because this can help to identify subjects who may need medical attention.

The finding that increasing age is a risk factor for COPD in nonsmokers is in line with previous studies results [22,26,27]. However, the relationship with gender is not entirely clear, indeed, some studies have found nonsmokers with airway obstruction to be predominately male [23,5,24], while others have found female sex to be a risk factor for COPD [34,35,36].

In the present study, the prevalence of COPD among nonsmokers was higher in the oldest old men than that seen in women and according to the multivariate analysis, male gender constitutes a statistically significant risk factor of airway obstruction. This result is consistent with findings from Germany, Norway, Philippines and Sweden [22–37] but in contrast with the results from Austria, South Africa, Iceland, Poland and Australia which showed a greater prevalence of COPD among female in comparison to male nonsmokers [22,35,36].

There is actually a considerable controversy as to whether women are at greater risk than men given an equal exposure to risk factors for COPD since they live longer than men and are more likely to be exposed to high indoor air pollution levels especially in developing countries [34–38]. There is a lot of debate in the current literature whether exposure to biomass cooking may be a risk factor for COPD, particularly in low income settings, however the evidence is contradictory and more studies are needed to elucidate the relationship between gender and COPD [25–39].

In our study population, all of the nonsmokers and 92.9% of the smokers with GOLD grade I+ COPD were previously undiagnosed. This huge gap between physician diagnosis of COPD and the presence of airflow obstruction defined by spirometry, suggests that COPD diagnosis based on symptoms may not be adequate. The lack of recognition and under-diagnosis of obstructive lung disease among nonsmokers is possibly a result of little knowledge on this condition and of a poor understanding and appreciation of risk factors other than smoking.

Our results also revealed that 30.4% of nonsmokers with significant non-reversible airway obstruction reported a prior physician’s diagnosis of asthma compared to only 14.3% of smokers (X2 = 16.47, p<0.01). In our material a previous diagnosis of asthma constitutes a statistically significant risk factor for COPD in nonsmokers. This result correlated well with the literature indicated that asthma plays a specific and important role in increasing the risk of airflow obstruction in nonsmokers [25,40,41]. Indeed, a previous SAPALDIA publication described this association extensively [40] and asthma has been recently found to be associated with COPD in nonsmokers [23,8,41]. These findings are supported by recent genetic association studies demonstrating that genetic nucleotide polymorphism variants appear to reduce the risk of both asthma and COPD in smokers [41,42,43].

Nonetheless, in the present study, the fact that concomitant asthma and symptoms were more prevalent in the nonsmoking group could theoretically explain the misdiagnosed COPD cases in patients with asthma and chronic obstruction. Indeed, despite the availability of consensus guideline diagnostic recommendations, diagnostic confusion between COPD and asthma are common. Additional tests and tools can be helpful in the differential diagnosis, including questionnaires specifically developed to discriminate between COPD and asthma and, in special cases, imaging studies.

In our study, exposure to environmental tobacco smoke was not associated with COPD in nonsmokers. Several researchers were also not able to identify passive smoking as a risk factor for COPD [23,4,44]. However the latter contrasts with a recent Chinese study which found an association between passive smoking exposure and airways obstruction in nonsmokers [24]. These conflicting results might be associated with the size of our study population.

In addition to well-documented causative factors for COPD in nonsmokers (such as age and gender), other factors may also have a role in the aetiology of the disease [4]. Of these factors, low BMI has attracted general attention. Indeed, COPD can be associated with a progressive loss of skeletal muscle mass and according to several studies, low BMI is an independent predictor of the risk of death [45–46]. Our data showed increased odds of COPD in nonsmokers with low BMI (<20 kg/m^2^). Moreover, in the non-smoking group, the association between BMI alterations and the presence of GOLD grade I+ COPD was more pronounced in men than women. The same results were reported by Lamprecht et al in 2011 showing increased odds of COPD in nonsmokers with a BMI<20kg/m^2^ [22]. Celli et al also found an increased likelihood of obstruction in nonsmokers with low BMI [45]. Given the cross-sectional nature of our data, we can-not discriminate whether low BMI precedes COPD or is rather a consequence of the disease.

The current authors also found a small, but statistically significant, association between COPD and occupational exposure to dusts, gases and fumes. Consistent associations have been previously observed between exposure to workplace agents and COPD in multiple high-quality epidemiological studies and an exposure-response gradient was demonstrated in several researches [5–46]. Biological plausibility is supported by data from experimental studies demonstrating the induction of chronic bronchitis and/or emphysema in animals after exposure to several agents associated with COPD in epidemiological studies eg, endotoxin, mineral dusts, sulfur dioxide and vanadium) have been shown to be capable of inducing pathologically defined chronic bronchitis in animal models [22,47,48,49]. Thus, it is interesting to note that in our study population, subjects who were exposed to occupational dusts, gases and fumes had significantly more respiratory symptoms than others; indeed, our results showed that 92% of nonsmokers exposed to occupational pollutants suffered frequently from chronic bronchitis. Several agents known to be associated with clinically defined chronic bronchitis in humans has been also shown to increase risk of chronic cough, lower FEV_1_, and lower FEV_1_/FVC independent of personal tobacco use of these cohorts.

Another type of indoor pollution arises from coal and biomass combustion. In the present study, 27% and 17% of nonsmokers have been exposed to biomass smoke and coal, respectively and 88% had poor ventilation in the kitchen; among nonsmokers with COPD, 39% were exposed to coal smoke and 84% had poor ventilation in the kitchen. Unlike in developed countries, indoor air pollution, including coal and biomass combustion, may contribute substantially to COPD especially for subjects without smoking habits [24,50,51,52]. However, in the present study, the correlation between levels of exposition and the prevalence of COPD didn’t reach the statistical significance level.

In the present study, nonsmokers with GOLD grade I+ COPD reported significantly higher amounts of co-morbidities (asthma, heart disease, hypertension and chronic bronchitis) than both smoking COPD and healthy nonsmokers. This again supports the arguments of several studies demonstrating that chronic airway obstruction in nonsmokers follows a similar course and involves co-morbid disease in either instance [53].

Airflow obstruction in nonsmokers is important for several reasons: 1) compared with smokers with airflow obstruction, symptoms are equally present, use of medication for respiratory disease is lower and respiratory care utilization tends to be higher. 2) Several diseases associated with COPD in smokers are also reported in nonsmokers with COPD. For example, Echave et al reported an association between low FEV1 and incidence of cardiovascular disease, independently of smoking [53]. Other studies found an increased risk of lung cancer for nonsmokers with COPD compared to those without COPD [54–55].

Strengths of this study include the total number of subjects included, exact spirometry and accurate mapping of risk factors. However, some limitations of the present research need to be acknowledged: reference equations for spirometric variables are not available for North Africa; BOLD uses the widely accepted prediction equations derived from the third US NHANES which may not ideally fit for all studied populations [56]. The use of a fixed threshold to define airways obstruction is associated with some extent of misclassification [57]. When we used the LLN instead of the fixed ratio, the proportion of nonsmokers among all cases of COPD remains, however, almost unchanged.

Moreover, some analysis were limited because of the low number of nonsmokers with COPD which limit our ability to draw conclusions regarding potential risk factors and because of the design of the questionnaires which were intended to be comprehensive and easy to administer but were based on self-reporting, and the latter can be subject to inaccurate recall.

## Conclusions

In this study, the prevalence, profiles and potential risk factors for GOLD grade I and higher COPD were elucidated among nonsmokers. The results revealed that nonsmokers constitute a substantial proportion of COPD patients, indeed, 45% of the participants with obstruction are nonsmokers, this prevalence appear to be at the higher range compared to other parts of the world with similar age distribution. Multiple risk factors, other than tobacco smoke, play a key role in the development and pathogenesis of COPD among nonsmokers as increasing age, male gender, lower BMI, exposure to occupational irritants and a prior diagnosis of asthma. The nonsmokers with airway obstruction had also significantly more symptoms and more co-morbid condition than both smoking COPD and healthy nonsmokers. Therefore, compared with the condition in smokers, airflow obstruction in non- smokers may arise from a different aetiology.
